# STK3 kinase activation inhibits tumor proliferation through FOXO1-TP53INP1/P21 pathway in esophageal squamous cell carcinoma

**DOI:** 10.1007/s13402-024-00928-8

**Published:** 2024-03-04

**Authors:** Ziying Zhao, Yuan Chu, Anqi Feng, Shihan Zhang, Hao Wu, Zhaoxing Li, Mingchuang Sun, Li Zhang, Tao Chen, Meidong Xu

**Affiliations:** Endoscopy Center, Department of Gastroenterology, Shanghai East Hospital, School of Medicine, Tongji University, Shanghai, 200120 China

**Keywords:** STK3/MST2, Hippo pathway, Esophageal squamous cell carcinoma, Apoptosis, Cell cycle arrest

## Abstract

**Purpose:**

Esophageal squamous cell carcinoma (ESCC) is an aggressive disease with a poor prognosis, caused by the inactivation of critical cell growth regulators that lead to uncontrolled proliferation and increased malignancy. Although Serine/Threonine Kinase 3 (STK3), also known as Mammalian STE20-like protein kinase 2 (MST2), is a highly conserved kinase of the Hippo pathway, plays a critical role in immunomodulation, organ development, cellular differentiation, and cancer suppression, its phenotype and function in ESCC require further investigation. In this study, we report for the first time on the role of STK3 kinase and its activation condition in ESCC, as well as the mechanism and mediators of kinase activation.

**Methods:**

In this study, we investigated the expression and clinical significance of STK3 in ESCC. We first used bioinformatics databases and immunohistochemistry to analyze STK3 expression in the ESCC patient cohort and conducted survival analysis. In vivo, we conducted a tumorigenicity assay using nude mouse models to demonstrate the phenotypes of STK3 kinase. In vitro, we conducted Western blot analysis, qPCR analysis, CO-IP, and immunofluorescence (IF) staining analysis to detect molecule expression, interaction, and distribution. We measured proliferation, migration, and apoptosis abilities in ESCC cells in the experimental groups using CCK-8 and transwell assays, flow cytometry, and EdU staining. We used RNA-seq to identify genes that were differentially expressed in ESCC cells with silenced STK3 or FOXO1. We demonstrated the regulatory relationship of the TP53INP1/P21 gene medicated by the STK3-FOXO1 axis using Western blotting and ChIP in vitro.

**Results:**

We demonstrate high STK3 expression in ESCC tissue and cell lines compared to esophageal epithelium. Cellular ROS induces STK3 autophosphorylation in ESCC cells, resulting in upregulated p-STK3/4. STK3 activation inhibits ESCC cell proliferation and migration by triggering apoptosis and suppressing the cell cycle. STK3 kinase activation phosphorylates FOXO1^Ser212^, promoting nuclear translocation, enhancing transcriptional activity, and upregulating TP53INP1 and P21. We also investigated TP53INP1 and P21’s phenotypic effects in ESCC, finding that their knockdown significantly increases tumor proliferation, highlighting their crucial role in ESCC tumorigenesis.

**Conclusion:**

STK3 kinase has a high expression level in ESCC and can be activated by cellular ROS, inhibiting cell proliferation and migration. Additionally, STK3 activation-mediated FOXO1 regulates ESCC cell apoptosis and cell cycle arrest by targeting TP53INP1/P21. Our research underscores the anti-tumor function of STK3 in ESCC and elucidates the mechanism underlying its anti-tumor effect on ESCC.

**Supplementary Information:**

The online version contains supplementary material available at 10.1007/s13402-024-00928-8.

## Introduction

Esophageal cancer is a significant public health concern, with a reported incidence of 604,000 cases and 544,000 deaths in 2020, ranking it seventh in incidence and sixth in mortality worldwide [1]. This type of cancer comprises two subtypes: esophageal adenocarcinoma (EAC) and esophageal squamous cell carcinoma (ESCC). ESCC is responsible for over 90% of esophageal cancer cases annually, with more than 80% occurring in eastern Asian regions, leading to high mortality rates and health burdens. Unfortunately, ESCC is a particularly formidable malignancy characterized by late-stage diagnosis, metastasis, therapy resistance, and frequent recurrence [2, 3]. ESCC is a devastating form of cancer often diagnosed at advanced stages, with frequent metastasis, resistance to therapy, and high recurrence rates [4].

ESCC can be classified into three major categories based on gene mutation status: 50 cases of ESCC1, 36 cases of ESCC2, and 4 cases of ESCC3 in a cancer genome atlas research. ESCC1 is characterized by dysregulation and abnormal expression of NFE2L2, SOX2/TP63, and YAP1 [5]. Studies have shown that YAP1 plays a significant prognostic role in ESCC and that YAP1 amplification is associated with lower survival rates in ESCC patients [6]. YAP/TEAD, a transcriptional component of the Hippo pathway, promotes the expression of genes linked to cell survival and proliferation when Hippo signaling is inactive.

STK3/4 kinases are highly conserved components of the Hippo pathway, regulating critical cellular processes such as immune responses, cell differentiation, organ regeneration, and tumor development [7, 8]. Structurally, STK3 and STK4 exhibit significant similarity and consist of two functional domains: an N-terminal kinase domain and a C-terminal regulatory region. Of particular note is a unique “SARAH” domain in their C-terminal region necessary for kinase activity. By interacting with their SARAH domains, STK3/4 forms homo- or heterodimers, leading to simultaneous phosphorylation at Thr180 for STK3 and Thr183 for STK4, thus activating the loop [9, 10]. In the classical Hippo pathway, STK3/4 facilitates the phosphorylation of LATS1/2, ultimately leading to the inactivation of YAP/TEAD by phosphor-LATS1/2. In contrast, when the Hippo pathway is inactive, YAP1/TEAD becomes underphosphorylated and enters the nucleus. In the nucleus, YAP1 interacts with the TEAD family of transcription factors or other elements, promoting the expression of genes associated with cell survival, proliferation, and metastasis [11, 12].

Numerous studies have shown that the level of STK3/4 phosphorylation remains low and does not undergo significant changes during the Hippo pathway activation, indicating that mammalian STK3/4 may not play a pivotal role in the Hippo pathway [13]. Due to the deletion of STK3/4, which leads to organ overgrowth and tumor advancement, it is reasonable to speculate about additional non-canonical roles of STK3/4. In contrast to Drosophila research models where Hpo/Stk is the only kinase that directly phosphorylates the hydrophobic motif of Wts/Lats, the hydrophobic motif of LATS can still be phosphorylated in mammalian models, even in STK3/4 knockout MEFs or hepatocytes, indicating that the Hippo pathway in mammals may be more complex and involves interactions with other pathways [14]. This suggests that the protein interaction network of STK3/4 may form a binding complex that implements various biological functions. Several research studies have elucidated the interaction network and various post-transcriptional modification (PTM) patterns of STK3 kinase in mammalian cells and its distinctive function, offering novel insights into this important kinase [15, 16]. Collectively, these findings enhance our understanding of the multifaceted activities of STK3 in biological processes and highlight its significance in various disease states, particularly cancer. The Hippo pathway is an evolutionarily conserved system in mammals, and the absence of STK3 has been shown to cause organ dysfunction and premature death in embryos. There have been limited studies demonstrating the crucial role of STK3 in negative tumor regulation, and further investigation is needed to determine its function in ESCC.

In this study, we focused on STK3 kinase, the upstream kinase of the Hippo pathway, to investigate its role and the mechanism in ESCC. Our findings suggest that STK3 kinase may suppress tumor growth through a non-canonical pathway. Oxidative stress activates the autophosphorylation of STK3, resulting in the upregulation of p-FOXO1^Ser212^ and the nucleus accumulation of FOXO1, which promotes the transcriptional activity of FOXO1 on the downstream genes TP53INP1 and P21. Amplification of STK3 led to tumor size suppression and cell apoptosis in ESCC, and the deletion of FOXO1 could reverse the effect.

## Materials and methods

### Cell culture and drug treatment

The cell lines utilized in this study were procured from the American Type Culture Collection (ATCC), and their authenticity was confirmed through specific indexes. Specifically, KYSE410, KYSE150, KYSE30, ECA109, and TE1 cell lines were cultured at 37 °C in a humidified 5% CO_2_ environment using either RPMI-1640 or DMEM supplemented with 10% FBS (Gibco), 100 U/ml penicillin, and 0.1 mg/ml streptomycin (Gibco). Hydrogen peroxide (H_2_O_2_) was freshly prepared by dilution in DMEM and employed at a predetermined final concentration (Thermo, 7722-84-1). N-Acetylcysteine was diluted to 5 mM concentration in ddH_2_O (Yeason, 50303ES05), while Cisplatin was diluted in ddH_2_O and employed at a different concentration (Yeason, 51401ES60).

### Plasmids and lentiviral infection

Small interfering RNA molecules were utilized to introduce targeted gene expression modulation in ESCC cell lines. These RNA molecules were transfected using the Hanbio reagent (HB-RF-1000). Additionally, plasmids containing the genes pET28a-FOXO1, pCMV-TP53INP1, and pEnCMV-P21 were transfected into ESCC cell lines via Lipofectamine™ 3000 Transfection Reagent (Thermo, L3000015). Furthermore, ESCC cell lines were transfected with recombinant lentiviruses, including pcDNA-STK3, LV-STK3-RNAi, and pLV2-CMV-FOXO1(S212R)-Puro, which was obtained from Genechem, Shanghai. The specific targeted sequences for these interventions can be found in Supplemental Table S1.

### Quantitative PCR analyses

Total RNA was isolated from cultured cells using the Trizol reagent (Invitrogen). To synthesize cDNA, we employed the PrimeScript RT reagent kit (TakaRa), followed by quantitative PCR with the qPCR SYBR Green Master Mix (Low Rox) (Yeason, Shanghai, 11202ES03) and primer pairs listed in Supplementary Table S2. Normalization to β-Actin mRNA allowed us to calculate the relative abundance of mRNA.

### Western blot and immunoprecipitation assay

The cultured cells were lysed in RIPA lysis buffer (Beyotime, P0013B) or by nuclear separation and cytoplasmic protein extraction using a kit (Beyotime, P0028), which was supplemented with proteinase and phosphatase inhibitors. The protein samples obtained from the cell lysates were processed by SDS-PAGE Loading Buffer (Beyotime, P0015L), then subjected to SDS-PAGE and transferred to a PVDF membrane (Millipore, #ISEQ00010). Following this, the membranes were blocked using 5% non-fat milk for one hour at room temperature, after which they were probed with primary antibodies against various targets. The antibodies used included STK3 (Abcam, ab52641), phospho-MST1(Thr183)/MST2(Thr180) (CST, #49332), FOXO1(CST, #2880), p-FOXO1^256^ (CST, #84192), p-FOXO1^212^ (ABclonal, customized antibody), TP53INP1(Abcam, Ab202026), P21 (Abcam, ab109520), BIM (ABclonal, A19702), active caspase 3 (ABclonal, A11021), 14-3-3-alpha/beta (ABclonal, A9151), and Lamin B1 (ABclonal, A11459), and β-Actin (ABclonal, AC036). Finally, the immunoreactive bands were quantified using an enhanced chemiluminescence Western blotting system (Tanon, Shanghai). For the immunoprecipitation assay, cells were cultured in 10 cm wells, and the cells were collected and lysed in RIPA with proteinase and phosphatase inhibitors. Then the cell lysates were incubated with A/G agarose (Santa Cruz, USA) and antibodies overnight, and the samples were processed with SDS-PAGE loading buffer.

### Immunofluorescence analysis and immunohistochemistry analysis

Transfected cells were subsequently seeded onto coverslips in 6-well plates for immunofluorescence analysis. Following two washes with phosphate-buffered saline (PBS), the cells were fixed with 4% formaldehyde for 20 min and then permeabilized with 0.1% Triton X-100 diluted in PBS for 2 min at room temperature. The processed coverslips were then incubated with primary antibodies overnight at 4 °C, followed by secondary antibody (2 h) and 1% DAPI (2 min) at room temperature. After being washed twice with 1% bovine serum albumin, the coverslips were mounted onto glass slides, and images were captured using a Leica Microsystems CMS GmbH microscope (TYPE DM6000B). For the immunohistochemistry (IHC) analysis, 100 slides of ESCC samples and 80 slides of adjacent tissues were subjected to routine pathological block preparation. The protein was blocked according to the instructions. The first antibodies of STK3 were applied on the slides overnight at 4 °C, and the biotinylated secondary antibody was incubated on the second day. Hematoxylin was used to counterstain, and a microscope was used to take photos of the slides. Two pathologists examined all slides and evaluated the immunostaining score according to intensity and percentage.

### CCK-8 assay and EdU staining

The proliferation of cells was assessed using the Cell Counting Kit-8 assay, which involved seeding 2000 cells in each well of a 96-well plate. After the cell adhesion, we incubated the cells with CCK8 reagent (10 μl reagent and 90 μl medium per well) for 1.5 hours. The cell proliferation was examined at 450 nm wavelength using a microplate reader in 24-h intervals for a duration of 5 days, following the manufacturer’s instructions. Additionally, the EdU staining was performed using the EdU Cell Proliferation Kit (Beyotime, C0078S). The transfected cells were cultured on coverslips with or without treatment, followed by incubation with EdU solution for 12 hours. The coverslips were washed with PBS and processed according to the instructions, and then the coverslips were fixed onto microscope slides and analyzed using a microscope (Leica, Germany, DM3000).

### Transwell assay and wound scratch assay

To assess the migratory capacity of ESCC cells, cells were transfected with specific plasmids, enumerated, seeded into individual chambers (Corning, 3422), and then incubated for 24–48 h, depending on the specific conditions. Following fixation and staining of the migrating cells, the number of migratory cells was quantified using a microscope. The extent of migration was expressed as the number of treated cells. For the wound scratch assay, cells were seeded into 6-well plates, and a scratch was created in the designated area at 0 and 24 h, respectively (48 h in ECA109 cell lines). The wound healing area was delineated using ImageJ (Fiji software) software.

### Chromatin immunoprecipitation (ChIP) assay

The DNA-binding profile of FOXO1 in KYSE150 and TE1 cell lines was investigated using the ChIP-IT High Sensitivity® kit (Active Motif) with the ChIP technique. Specifically, the chromatin samples from sheared cells were immunoprecipitated using FOXO1 antibody and IgG as negative control at 4 °C overnight. Subsequently, the ChIP-purified DNA was quantified using Q-PCR and further confirmed by agarose gel (3%) electrophoresis analysis. The primer sets for Q-PCR were listed in Supplemental Table S3.

### In vivo xenograft mouse model

KYSE150 cells were genetically modified to express shRNA-STK3 target 1 and pcDNA-STK3 or negative control and then cultured. The collected cells were injected subcutaneously (7 × 10^6^ cells in each group, diluted in 1 ml PBS) into the right armpit region of 6-week-old female athymic nude mice, each group comprising 4 mice. Tumor formation was observed at 5-day intervals, and on the 30th day, the mice were euthanized, and the subcutaneous tumors were examined. A portion of the tumor was fixed in 3.7% formaldehyde, embedded in paraffin, and sliced for hematoxylin and eosin staining and histological analysis. Tumor volume was calculated using the formula V = Width × Width × Length × 0.5 (mm^3^). All animal experiments were conducted by the guidelines of the Johns Hopkins University Animal Care and Use Committee.

### Flow cytometric analysis

The ROS level was measured using the Reactive Oxygen Species Assay Kit (Beyotime, S0033S), following the manufacturer’s instructions. Specifically, cells were treated with H_2_O_2_ and then incubated with DCFH-DA at 37 °C before fluorescence distribution was analyzed using flow cytometry. Additionally, cells transfected with specific plasmids were stained with propidium iodide (PI) and analyzed using the Cell Cycle and Apoptosis Analysis Kit (Beyotime, C1052) on the flow cytometer for cell cycle analysis. To assess apoptosis, transfected cell lines were stained with PI and Annexin V-FITC (or Annexin V-APC) using the Annexin V-FITC/PI Apoptosis Kit (Elabscience) or the Annexin V, 633 Apoptosis Detection Kit (Dojindo) in accordance with the manufacturer’s instructions. Flow cytometry was employed in all three assays, and each tube contained a minimum of 10,000 stained cells. Data were collected from three independent experiments and analyzed using FlowJo analysis software.

### ATP measurement

Various experimental conditions were applied to the cells, after which the cellular ATP levels were determined. The collection of cell lysates was followed by the measurement of ATP levels using the Enhanced ATP Assay Kit (Beyotime, Shanghai) and quantified through luminometry utilizing Spectrum 5. The standard curve was used to determine the ATP levels.

### Statistical analysis

GraphPad Prism 9 and Microsoft Excel 2019 were employed for conducting data analyses. Student’s t-test was used to compare two groups, whereas ANOVA tests were used to compare three or more groups. The Kaplan-Meier curve was used for evaluating the overall survival. The data were derived from three independent experiments and expressed as means ± standard error (S.E.). Statistical significance was described: *p < 0.05, **p < 0.01, ***p < 0.001, ****p < 0.0001, while “ns” indicated a lack of significance.

## Results

### ESCC samples showed significantly higher expression of STK3 compared to normal esophageal epithelium

**Fig. 1:**
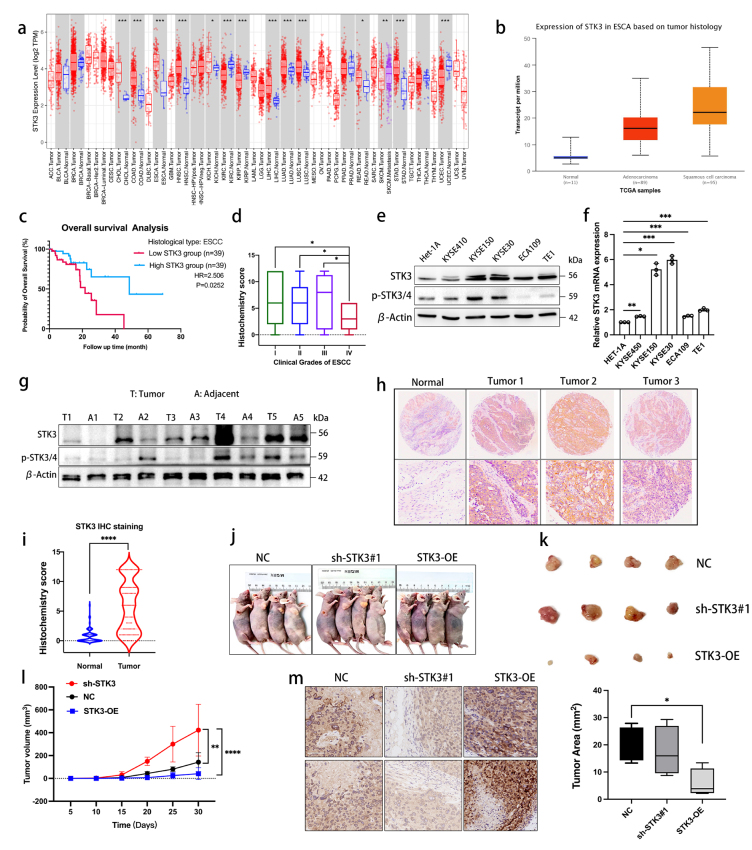
ESCC samples showed significantly higher expression of STK3 compared to normal esophageal epithelium. (**a**) Bioinformatics data showed the expression difference of STK3 in cancers. (**b**) Bioinformatics analysis of the expression level of STK3 in ESCC, EAC, and normal esophageal squamous tissue. (**c**) Kaplan-Meier curve was made to evaluate the survival probability in ESCC patients according to the expression level of STK3. The data were collected from the TCGA database. (**d**) The patients’ data from our ESCC clinical database were divided into four groups by the clinical stages (diagnosed according to AJCC8), and the relevance was investigated. (**e, f**) Het-1A cells representing normal esophageal squamous epithelium, along with ESCC cell lines KYSE450, KYSE150, KYSE30, ECA109, and TE1 cells, were cultured under standard conditions; the cell lysates were subjected to Western blotting using the specific antibodies. Total RNA was extracted and subjected to Q-PCR analyses for steady-state mRNA levels of STK3. Data were presented from three independent experiments in duplicate. (**g**) Five pairs of ESCC samples and adjacent tissue lysates were subjected to Western blotting. (**h, i**) 100 pieces of ESCC samples and 80 normal esophageal epithelium were assessed via IHC. (**j, k**) The xenograft model of nu mice and representative image of fresh tumor tissue (n = 4 mice per group). (**l**) The tumor size quantification results were documented for 30 days at the 5-day interval (**p < 0.01, ****p < 0.0001.). (**m**) IHC analysis of tumor tissues from three groups. The tumor area was evaluated by AI assistant analysis (*p < 0.05)

In this study, we utilized data from TCGA to calculate the expression levels of STK3 in ESCA using online software (http://ualcan.path.uab.edu/analysis.html) (Fig. 1a, b). To further elucidate the functional implications of STK3 and in ESCC, we downloaded data from the TCGA website (https://portal.gdc.cancer.gov/projects/TCGA-ESCA) and generated Kaplan-Meier curves to evaluate overall survival based on STK3 expression levels (Fig. 1c). The patients’ data achieved from our ESCC clinical database were divided into four groups according to the clinical stages (The 8th Edition of the American Joint Committee on Cancer (AJCC8) Staging Manual), and we found the IHC score difference of STK3 between early stage and the late stage of ESCC (Fig. 1d).

Furthermore, we investigated the relative protein and mRNA expression levels of STK3 in various ESCC cell lines, including KYSE150, KYSE450, KYSE30, ECA109, and TE1, and compared to normal esophageal epithelium cell line HET-1A, by using Western blotting (Fig. 1e) and quantitative PCR (Fig. 1f), respectively. To determine the abundance of STK3 in ESCC tissue samples relative to normal esophageal epithelium tissue, we examined the protein expression levels of STK3 and p-STK3/4 using Western blotting on protein samples obtained from five pairs of ESCC tissues and normal adjacent epithelium tissues (Fig. 1g). We also investigate the expression levels of STK3 in ESCC tissue in comparison to normal tissue, utilizing IHC (Fig. 1h, i).

To investigate the impact of STK3 on ESCC tumor growth in vivo, a nude mouse model was constructed via injection of KYSE150 cells from negative control, sh-STK3#1, and STK3-OE groups. The results demonstrated that the deletion of STK3 expression substantially promotes tumor progression and augments tumor size. Conversely, overexpression of STK3 impeded tumor progression (Fig. 1j–l). The histological evaluation indicated variable levels of STK3 expression in the three experimental groups (Fig. 1m).

### STK3 regulates in vitro ESCC cell proliferation, migration, and chemoresistance

**Fig. 2:**
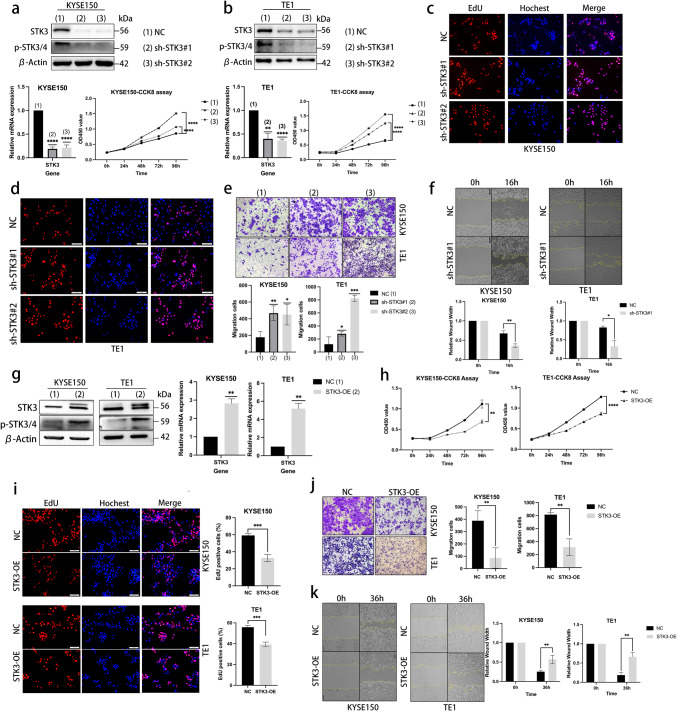
STK3 regulates in vitro ESCC cell proliferation, migration, and chemoresistance (**a, b**) KYSE150, TE1 cells stably expressing shRNA specific to STK3 were subjected to Western blotting, and quantitative PCR was shown. The CCK8 assay was presented as well. (**c, d**) EdU assay showed that EdU-positive cells increased according to the STK3 deletion. (**e**) KYSE150, TE1 cells stably expressing shRNA specific to STK3 were incubated for 36 h and then subjected to transwell assay. Migrated cells were fixed by 4% formalin and stained with crystal violet. Scale bar = 100 μm. (**f**) KYSE150 and TE1 cells stably expressing shRNA-STK3 were seeded in the 6-well tissue culture plates, and wound width of KYSE150 and TE1 cells with or without STK3 stably deletion at 0 and 16 h were measured. (**g**) KYSE150, TE1 cells stably expressing pcDNA-STK3 (STK3-Overexpression, STK3-OE) were cultured, and cell lysates were subjected to Western blotting and Q-PCR analysis. (**h, i**) KYSE150, TE1 cells transfected with pcDNA-STK3 were cultured and subjected to CCK8 assays and Eud assay. (**j**) Transwell assay showed the migration cells decreased in the STK3-OE group. (**k**) KYSE150 and TE1 cells with or without STK3 overexpression were seeded in 6-well plates, and wound width was measured at 0 and 36 h separately (Data are presented as the means ± standard error (S.E.). (*p < 0.05, **p < 0.01, ***p < 0.001, ****p < 0.0001)

In this part, we investigated the functional role of STK3 kinase in ESCC cell lines. Specifically, we explored the effect of STK3 knockdown and overexpression on various cellular processes, including proliferation and migration. Our results demonstrated that STK3 could be activated at the Thr180 site and form hetero- or homo-dimerization with phosphor-STK3 (Thr180) or phosphor-STK4 (Thr183). To investigate the functional relevance of STK3 in ESCC, we silenced STK3 gene using shRNA loaded by lentivirus and confirmed the efficacy of knockdown through western blotting and quantitative PCR in KYSE150, TE1, and ECA109 cell lines. The CCK8 assay showed a significant reduction in proliferation rates in the knockdown group compared to the NC group (Figs. 2a, b, and S1d). EdU assay revealed that STK3 deletion led to an increase in EdU-positive cells, indicating an association between STK3 deletion and cell proliferation in KYSE150 and TE1 cell lines (Figs. 2c, d, and S1a). Furthermore, we found a positive correlation between STK3 knockdown and increased cell migration through transwell and wound scratch assays (Figs. 2e, f, and S1e, f).

To determine the effect of STK3 overexpression on ESCC, we transfected STK3-targeting pc-DNA to express high STK3 levels in three ESCC cell lines (Figs. 2g and S1g). Our results demonstrated that STK3 overexpression inhibited the proliferation and migration phenotype of ESCC cells, as confirmed by CCK8, transwell assays, and wound scratch assay (Figs. 2h, j, k, and S1h, i). EdU-positive cells decreased significantly as well (Fig. 2i). Drug resistance to cisplatin was also measured. In two cell lines, the IC50 values of the shSTK3#1 group were statistically significantly higher than those of the NC group, suggesting increased drug resistance to cisplatin (Fig. S1b). Additionally, STK3-overexpressed cells exhibited an increased sensitivity to cisplatin (Fig. S1c). In summary, our data suggest a potential inhibitory role of STK3 in ESCC proliferation, viability, and cell migration.

### A moderate level of cellular ROS induces autophosphorylation of STK3, correlating with tumor suppression

**Fig. 3:**
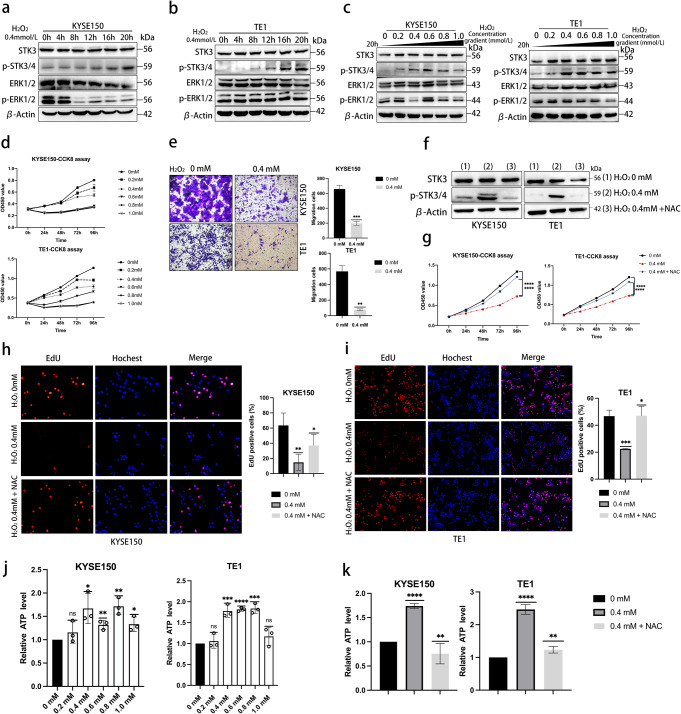
A moderate level of cellular ROS induces autophosphorylation of STK3, correlating with tumor suppression. (**a, b**) KYSE150 and TE1 cells were exposed to 0.4 mM H_2_O_2_ diluted in DMEM. Cell lysates were collected at 4-hour intervals and analyzed by Western blotting with specific antibodies. (**c**) KYSE150 and TE1 cells were treated with different concentrations of H_2_O_2_ (0–1.0 mM) for 20 h, and the cell lysates were subjected to Western blotting. (**d**) KYSE150 and TE1 cells were cultured for 24 h in 96-well plates, then treated with H_2_O_2_ (0, 0.2, 0.4, 0.6, 0.8, 1.0 mM) and subjected to CCK8 assays. (**e**) For transwell assay, cells were seeded in the 24-well chambers and treated with 0 or 0.4 mM H_2_O_2_ for 24 h. Migrated cells were fixed by 4% formalin and stained with crystal violet. Scale bar = 100 μm. (**f, g**) KYSE150 and TE1 cells were treated with H_2_O_2_ (0.4 mM) with or without NAC (5 mM, diluted in ddH_2_O) for 24 h, and the cells were subjected to the CCK8 assay. The lysates were subjected to Western blotting to evaluate the protein phosphorylation level. (**h, i**) The Same culture conditions for the EdU assay and the EdU-positive cells were measured between groups; analysis was shown as the means ± S.E. from three independent experiments. (**j**) KYSE150 and TE1 cells were treated with different concentrations of H_2_O_2_, and the cell suspension was processed and measured according to the manufacturer’s instructions. (**k**) KYSE150 and TE1 cells were treated with H_2_O_2_ with or without NAC, and all samples were processed according to the instructions and were subjected to the luminometer. (*p < 0.05, **p < 0.01, ***p < 0.001, ****p < 0.0001.)

Autophosphorylation of STK3 induced by H_2_O_2_ was further investigated. The findings revealed that a moderate concentration of H_2_O_2_ could elevate cellular ROS levels, consequently promoting the time-dependent upregulation of p-STK3/4 occurring within 20 h (Fig. S2a, b). Within 20 h, a concentration of 0.4 mM H_2_O_2_ was found capable of inducing autophosphorylation of STK3 in both KYSE150 and TE1 cells. Conversely, phosphor-ERK1/2, a classic phosphorylation protein known for its pro-cancer role, did not display any noticeable augmentation in the H_2_O_2_ stimuli model (Fig. 3a, b). Moreover, the concentration gradient model showed that the expression of p-STK3/4 was significantly upregulated at 0.4 mM H_2_O_2_ (Fig. 3c), and H_2_O_2_ treatment further elevated the p-STK3/4 level in STK3-OE groups (Fig. S2c). We used flow cytometry to examine the ROS level in the H_2_O_2_ concentration gradient model. Treatment with a specific concentration of H_2_O_2_ improved the FITC positive cell proportion, indicating a concentration-dependent increase of ROS level in the H_2_O_2_ concentration gradient model (Fig. S2d, f). In the rescue experiment, N-acetylcysteine (NAC) was used to eliminate intracellular ROS, and western blotting was performed to determine the expression level of phosphor-STK3/4 (Fig. 3f). The flow cytometry results showed that NAC treatment could reduce the proportion of FITC-positive cell (Fig. S2e, g). Overall, the results indicated that a moderate concentration of H_2_O_2_ treatment could induce cellular ROS accumulation and the autophosphorylation of STK3 in KYSE150 and TE1 cells.

Furthermore, cell viability was assessed at different concentrations of H_2_O_2_, revealing that 0.4 mM was a moderate concentration for ESCC cell lines’ growth, while high concentrations proved lethal to the cells (Fig. 3d). The study then investigated the migration ability of KYSE150 and TE1 at the moderate H_2_O_2_ concentration of 0.4 mM. Both cell lines exhibited a significant decrease in migration proportion in the 0.4 mM H_2_O_2_ stimulation group, consistent with the upregulation of p-STK3/4 (Fig. 3e). The NAC rescue group was also observed to efficiently reverse the suppressive effect of H_2_O_2_ on ESCC cell proliferation, as shown in the CCK8 assays (Fig. 3g) and EdU assay (Fig. 3h, i). These findings suggest that H_2_O_2_ treatment can regulate ESCC cell viability and migration ability through the induction of ROS accumulation and the autophosphorylation of STK3.

Adenosine triphosphate (ATP) is a critical supplier of phosphate groups essential for the dimerization of serine/threonine kinase. To investigate further the mechanism of STK3 autophosphorylation under H_2_O_2_-induced oxidative stress, we examined cellular ATP levels in varying H_2_O_2_ concentrations. Our findings suggest that there exists a potential correlation between cellular ROS and an increased ATP level, while higher levels of H_2_O_2_ appear to impair the activity of H+-ATP synthase, thereby resulting in a reduction in ATP levels (Fig. 3j, k) [17].

It has been reported that elevated H_2_O_2_ levels can disrupt cellular processes by triggering necrosis and apoptosis. Consequently, we have identified STK3 as a crucial kinase in the Hippo pathway, showing a significant relationship between its phosphorylation state and kinase activity. Therefore, the concentration of H_2_O_2_ mentioned earlier (0.4 mM) was deemed appropriate for modifying the function of STK3, making it a recommended protocol for other researchers.

### Activated STK3 coactivate with FOXO1 and phosphorate FOXO1 at forkhead domain Ser^212^

**Fig. 4:**
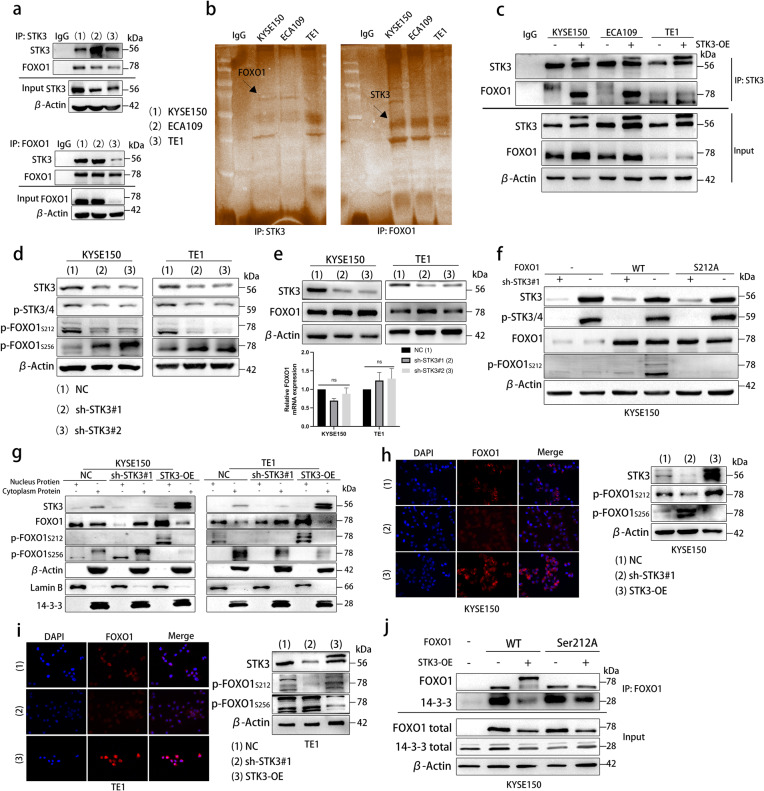
Activated STK3 coactivate with FOXO1 and phosphorate FOXO1 at forkhead domain Ser^212^ (**a**) Immunoprecipitation samples and Input/IgG samples of STK3 and FOXO1 in KYSE150, ECA109, and TE1 cells, the lysates were subjected to the Western blotting. Immunoglobulin-G (IgG) was used as the negative control, and Input as a positive control. (**b**) The STK3 and FOXO1 immunoprecipitation samples were subjected to Western blotting. The gels were processed according to the silver staining instructions. (**c**) IP samples of KYSE150, ECA109, and TE1 cells with or without STK3 overexpression were subjected to Western blotting using the specific antibodies. IgG was used as the negative control, and Input as the positive control. (**d**) KYSE150 and TE1 cells stably expressing shRNA-STK3 were subjected to western blotting, and the p-FOXO1^212^ and p-FOXO1^256^ expressions were specially measured. (**e**) The total protein and mRNA expression of FOXO1 were evaluated (ns, not significant). (**f**) The KYSE150 cells with or without STK3 deletion were transfected with siRNA-FOXO1#1 and pVL2-CMV-FOXO1^S212A^. The cell lysates were subjected to western blotting using specific antibodies. (**g**) KYSE150 and TE1 cells stably transfected with the empty vehicle, shRNA-STK3, or pcDNA-STK3 were cultured in 6 cm plates for at least 24 h. The cytoplasm and nucleus proteins were extracted according to the manufacturer’s instructions. β-Actin and Lamin B were used respectively as the cytosolic and nuclear marker proteins to ensure the validity of our protocol. (**h, i**) KYSE150 and TE1 cells stably transfected with NC, shRNA-STK3 or pcDNA-STK3 were cultured in 6-well plates with or without coverslips. We collected the cell lysates and subjected them to Western blotting. We processed the coverslips according to the immunofluorescence staining protocol and then used the microscope to record the fluorescence distribution of FOXO1. (**j**) The KYSE150 cells with or without STK3-OE were transfected with siRNA-FOXO1#1, and pVL2-CMV-FOXO1^S212A^ were cultured in 10 cm plates, and the cell lysates using an anti-FOXO1 antibody for immunoprecipitation

While previous studies have identified FOXO1/FOXO3 as downstream substrates of STK4, the function of STK3, despite its unique role as a homolog kinase, has received limited attention. Our study aimed to investigate the interaction between STK3 and FOXO1 and the potential phosphorylation site of FOXO1 by STK3. Using immunoprecipitation analysis and western blotting, we demonstrated that STK3 interacts with endogenous FOXO1 in three ESCC cell lines (Fig. 4a). To confirm this interaction, silver staining was used to detect interaction proteins of IP samples (Fig. 4b). The protein bands were observed at the target position, which is consistent with the molecular weight of the interaction protein. Overexpression of STK3 was found to enhance the interaction between STK3 and FOXO1 in KYSE150, ECA109, and TE1 cell lines (Fig. 4c). We also used online analysis software to identify several important reported phosphorylation sites (serine 212 site and serine 256 site) of FOXO1 at a different domain (Fig. S3a).

Deletion of STK3 disrupted the competitive phosphorylation of FOXO1 at the Serine212 site, leading to an elevation in p-FOXO1^Ser256^, a site typically phosphorylated by Akt kinase (Fig. 4d). We also examined the total FOXO1 protein and FOXO1 mRNA in the sh-STK3#1, sh-STK3#2 and NC groups. The results showed that deletion of STK3 did not affect the total protein nor mRNA expression of FOXO1 (Fig. 4e). We further identified the phosphorylation relation of STK3 kinase on FOXO1 by transfecting FOXO1 mutant plasmids of arginine to replace serine at the 212 site in KYSE150 and TE1 cell lines (STK3 deletion group and NC group separately). This revealed a significant downregulation of p-FOXO1^Ser212^ in FOXO1^Ser212A^ and FOXO1 knockdown group, compared to FOXO1^wt^ in the NC group (Figs. 4f and S3b). Thus, we successfully demonstrated that FOXO1 could interact with STK3 and be phosphorylated by STK3 at the Serine^212^ site. Mutation of FOXO1 Serine212 using its corresponding mutant plasmids could abrogate the level of p-FOXO1^Ser212^ in parallel.

Previous research demonstrated that FOXO1 and FOXO3 could translocate from the nucleus to the cytoplasm in response to Akt phosphorylation at Serine^256^(FOXO1)/Serine^253^(FOXO3) and FOXO1/FOXO3 subsequent binding with 14-3-3 protein. The competitive inhibition of the phosphorylation on Serine^256^(FOXO1)/Serine^253^(FOXO3) could reverse the shuttle [18–20]. In the present study, we provide evidence that FOXO1 undergoes translocation from the cytosol to the nucleus in response to the activation of STK3. In this study, we conducted nuclear and cytoplasmic extraction experiments to investigate the distribution of FOXO1 in various test groups. Our results indicate that FOXO1 translocates to the nucleus in the STK3 overexpression group with an upregulating trend of p-FOXO1^Ser212^, in contrast to the sh-STK3#1 group or NC group (Fig. 4g). Immunofluorescence staining was employed visually display the distribution of FOXO1. As shown in Fig. 4h, i, upregulation of STK3 resulted in the accumulation of endogenous FOXO1 in the nucleus, whereas in the STK3 deletion group, the fluorescence of FOXO1 was dispersed in the cytoplasm. We conducted a co-immunoprecipitation assay to further elucidate the mechanism. Our results demonstrate that STK3 overexpression facilitates the separation of 14-3-3 and FOXO1. We also found that invalidation of FOXO1^Ser212^ inhibits the phosphorylation function of STK3 kinase on FOXO1, which promotes FOXO1 combination with 14-3-3 and export to the cytoplasm (Fig. 4j and S3c). These findings suggest that STK3 plays a role in regulating the distribution of FOXO1 through its phosphorylation of FOXO1^Ser212^ and the non-engagement between FOXO1 and the 14-3-3 protein. Notably, the translocation of FOXO1 to the nucleus may augment its transcriptional function, while its translocation to the cytoplasm may attenuate this effect.

### FOXO1 inhibits tumor progression and promotes apoptosis in ESCC

**Fig. 5:**
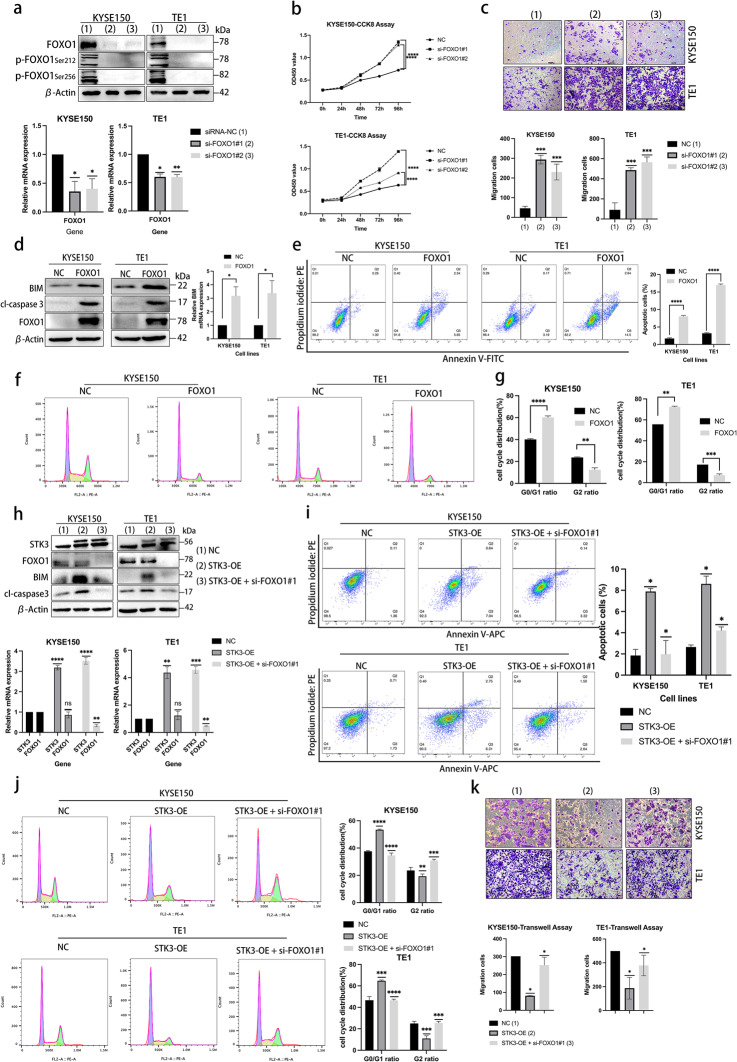
FOXO1 inhibits tumor progression and promotes apoptosis in ESCC. (**a**) KYSE150 and TE1 cells were transfected with siRNA-FOXO1, and protein and mRNA levels were assessed through western blotting and Q-PCR. (**b, c**) Cell viability and migration were evaluated by CCK8 assay and transwell assay (***p < 0.001, ****p < 0.0001.). (**d**) FOXO1 was overexpressed by pET28a-FOXO1 transfection in KYSE150 and TE1 cells, and cell lysates were subjected to western blotting by using specific antibodies. (**e**) KYSE150 and TE1 cells overexpressing FOXO1 were cultured for 48 h, and the supernatants and cells were collected and washed with PBS. The cell pellets were stained by propidium iodide and Annexin V-FITC and incubated for 15 min; then, the cell suspension was subjected to a flow cytometer for the apoptosis analysis. (**f, g**) KYSE150 and TE1 cells overexpressing FOXO1 were cultured for 48 h, and the cells were collected and washed with PBS, and incubated in 70% ethanol overnight. The cell lysates were washed by PBS the next day, and processed with propidium iodide stain for 30 min. The cell suspension was subjected to a flow cytometer for the cell cycle analysis. (**h**) In the rescue experiments, the cells expressing pcDNA-STK3 were transfected with or without siRNA specific to FOXO1. The protein and mRNA were examined by western blotting and Q-PCR. (**i**) In the rescue experiments, the cells transfected with the targeted gene were processed and subjected to a flow cytometer for apoptosis analysis. (**j**) In the rescue experiments, the cells transfected with the targeted gene were processed and subjected to a flow cytometer for cell cycle analysis. (**k**) The transfected cells were seeded in the 24-well chamber for the transwell assay (*p < 0.05, **p < 0.01, ***p < 0.001.)

The FOXO subfamily is involved in various critical cellular processes in mammalian cells, including apoptosis, stress resistance, cell cycle arrest, DNA damage repair response, and glucose metabolism, as evidenced by previous studies [21]. To investigate the specific role of FOXO1 in ESCC cell lines, we used a specific siRNA to knock down FOXO1 expression. We then utilized western blotting and Q-PCR to assess the effects of FOXO1 knockdown on protein and gene expression (Figs. 5a and S3d). The impact of FOXO1 knockdown on ESCC cell proliferation and migration was evaluated using CCK8 and transwell assays (Figs. 5b, c and S3e).

The pET28a-FOXO1 and empty vehicle were transfected into the KYSE150 and TE1 cell lines to investigate the effects of FOXO1 amplification. The results showed that FOXO1 amplification increases the apoptosis cell in both cell lines and upregulates the downstream pro-apoptotic proteins BIM and cleaved caspase 3 (Fig. 5d, e). The cell cycle assay was also conducted, revealing an increased G0/G1 ratio in the FOXO1 overexpression group. This observation suggests that FOXO1 plays a crucial role in inducing cell cycle arrest (Fig. 5f, g). Further western blotting and Q-PCR results from rescue experiments demonstrated that the overexpression of STK3 enhanced the anti-tumor function of FOXO1, evident in increased levels of BIM and cleaved-caspase 3. The deletion of FOXO1 in STK3 overexpression cells could reverse the downstream gene expression (Fig. 5h). Overexpression of STK3 increased the proportion of early and late apoptotic cells, which could be rescued by FOXO1 knockdown (Fig. 5i). The cell cycle assay revealed a consistent outcome, wherein the G0/G1 ratio demonstrated a notable increase in the STK3-OE group. Conversely, the introduction of FOXO1 knockdown was observed to reverse the cell cycle arrest (Fig. 5j). The results of the transwell assay indicated that the decreased migration effect of STK3 overexpression on ESCC cell lines could be reversed by FOXO1 deletion (Fig. 5k).

### STK3 activates the FOXO1-TP53INP1/P21 axis

**Fig. 6:**
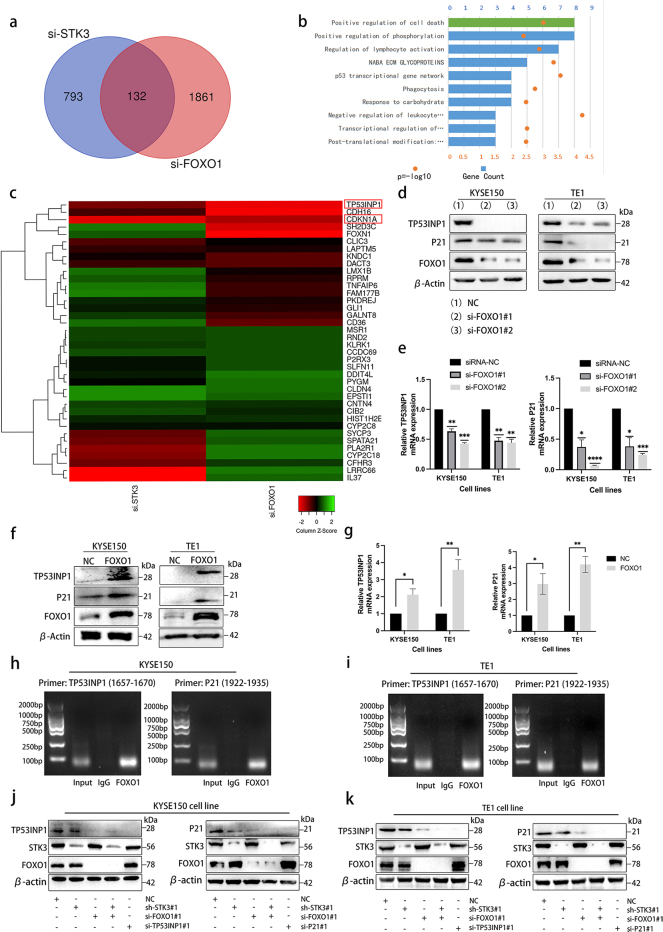
STK3 activates the FOXO1-TP53INP1/P21 axis. (**a**) Venn diagram was made according to the RNA-Seq results. (**b**) The top 10 Gene Ontology biological processes were obtained via online bioinformatics software, and the Pathway and Process Enrichment analysis was exhibited. (**c**) The overlapping part of differentially expressed genes of si-STK3 and si-FOXO1 were presented in a heatmap format. (**d, e**) KYSE150 and TE1 cells were transfected with siRNA specific to FOXO1, and cell lysates were subjected to western blotting and Q-PCR. (**f, g**) FOXO1 was overexpressed by transfecting pET28a-FOXO1 in KYSE150 and TE1 cells, and cell lysates were subjected to western blotting and Q-PCR. (**h, i**) The ChIP assay showed that the promoter fragments were shown as amplified DNA bands. (**j, k**) KYSE150 and TE1 cells were transfected with the empty vehicle, and the cells stably expressing sh-STK3 were transfected with or without si-FOXO1, and the cells transfected with si-FOXO1 or si-TP53INP1 or si-P21. All the cell lysates were immunoblotted with the indicated antibodies

Despite our demonstration that FOXO1 is a substrate of STK3 kinase, the downstream effector remains unknown. To explore this, we used RNA sequencing and overlapped the commonly differentially-expression genes of STK3 kinase and transcription factor FOXO1 using an online tool (http://bioinformatics.psb.ugent.be/), and we found 132 genes among the overlapping region (Fig. 6a). After mapping all differentially expressed genes to terms in a database, we focused on the gene related to positive regulation of the cell death pathway (https://metascape.org/) (Fig. 6b). The presented findings demonstrate that the expression of TP53INP1 (Tumor protein p53-induced nuclear protein 1) and CDKN1A (Cyclin-dependent kinase inhibitor 1, also known as P21) was significantly reduced in both si-STK3 and si-FOXO1 groups, as indicated by the heatmap (Fig. 6c). According to the previous study, TP53INP1 and P21 might be the critical transcriptional downstream gene of the FOXO1 [22]. Subsequently, we identified TP53INP1 and P21 as the putative downstream effectors of STK3/FOXO1. Western blotting and quantitative PCR analyses showed a significant reduction in TP53INP1 and P21 expression levels in the FOXO1 deletion group (Fig. 6d, e), and their expression levels were substantially downregulated by FOXO1 knockdown. Conversely, the overexpression of FOXO1 resulted in a concomitant upregulation of TP53INP1 and P21 (Fig. 6f, g), which suggested that the expression of TP53INP1 and P21 are closely regulated by of FOXO1. We utilized chromatin co-immunoprecipitation with antibodies to further demonstrate the regulatory relations. The results supported the transcriptional relationship of FOXO1 and TP53INP1/P21 in ESCC cell lines (Fig. 6h, i).

The downregulation of TP53INP1 and P21 was also found to be associated with the deletion of STK3, and the double knockdown of STK3 and FOXO1 resulted in significantly reduced expression levels of TP53INP1 and P21 in KYSE150 and TE1 cell lines (Fig. 6j, k). Taken together, these results highlight the crucial role of FOXO1 in mediating the transcription of TP53INP1 and P21 in ESCC cell lines. STK3 deletion significantly weakened the FOXO1 transcriptional function in ESCC, leading to a downregulation of downstream genes.

### TP53INP1 and P21 act as antitumor effectors mediated by FOXO1 in ESCC cell lines

**Fig. 7:**
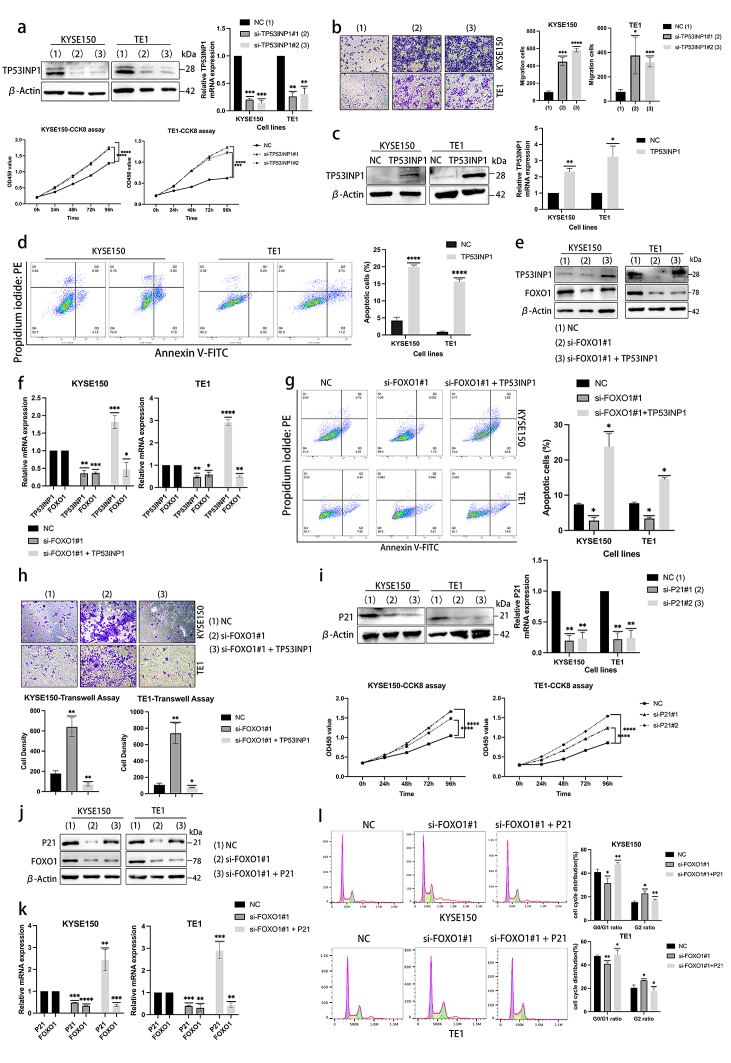
TP53INP1 and P21 act as antitumor effectors mediated by FOXO1 in ESCC cell lines. (**a**) KYSE150 and TE1 cell lines were transiently transfected with siRNA-TP53INP1, and the cell lysates were subjected to western blotting and Q-PCR. The cell viability was also examined by CCK8 assay. (**b**) Transwell assay of KYSE150 and TE1 cells transfected with siRNA specific to TP53INP1 (*p < 0.05, ***p < 0.001, ****p < 0.0001.). (**c**) The expression of TP53INP1 was upregulated by transfecting pCMV-TP53INP1 in KYSE150 and TE1 cell lines. The cell lysates were subjected to immunoblotting and Q-PCR. (**d**) The apoptosis analysis of TP53INP1 overexpression was examined in ESCC cell lines. (**e, f**) In the rescue experiment, the siRNA-FOXO1 was transfected singly or co-transfected with pCMV-TP53INP1 into the KYSE150 and TE1 cells. The cell lysates were subjected to western blotting and Q-PCR. (**g, h**) The rescue experiment under the same transfection conditions analyzed the apoptosis analysis and transwell assay from three independent tests (*p < 0.05, **p < 0.01.). (**i**) P21 was knocked down by specific siRNA in KYSE150 and TE1 cells, and the cell lysates were subjected to western blotting and Q-PCR. The cell viability was assessed by CCK8 assay. (**j, k**) A rescue experiment was shown, and the siRNA-FOXO1 was transfected singly or co-transfected with pEnCMV-P21. The cells were cultured for 48 h; then, cell lysates were subjected to immunoblotting and Q-PCR analysis. (**l**) In the rescue experiment under the same condition, the cells were processed according to the manufacturer’s instructions. The cell cycle profile was obtained from one of three independent experiments. The Flowjo analysis software analyzed the data (*p < 0.05, **p < 0.01.)

The protein TP53INP1 is recognized as having antiproliferative and proapoptotic properties, playing a role in the cellular stress response. To investigate the impact of TP53INP1 in KYSE150 and TE1 cells, we used western blotting and Q-PCR experiments to verify the knockdown efficiency of specific siRNA-TP53INP1. We also testified the function of TP53INP1 by CCK8 assay (Fig. 7a). Transwell assay was used to measure the migration cells between the NC and si-TP53INP1 group (Fig. 7b). Additionally, we conducted flow cytometric analysis to demonstrate the pro-apoptotic function of TP53INP1 (Fig. 7c, d). As aforementioned, we performed rescue experiments to elucidate further the direct involvement of TP53INP1 in FOXO1 activation (Fig. 7e, f). Our results showed that transfecting FOXO1-specific siRNA reduced the proportion of apoptotic cells in ESCC cell lines. However, transfecting both pCMV-TP53INP1 and si-FOXO1 reversed these effects, promoting cell apoptosis and inhibiting migration (Fig. 7g, h).

P21, as a mediator of cellular proliferation inhibition in response to DNA damage regulated by p53/TP53. In this study, we examined the effect of specific siRNA on P21 in KYSE150 and TE1 cells using western blotting and quantitative PCR. CCK8 assay showed the function of P21 on cell proliferation (Fig. 7i). Subsequently, a rescue experiment was conducted, where overexpression of P21 using pEnCMV-P21 restored the cell cycle progression that was previously induced by FOXO1 deletion (Fig. 7j–l). These findings suggest that P21 overexpression could impede the cell cycle and suppress proliferation in ESCC cell lines.

**Fig. 8:**
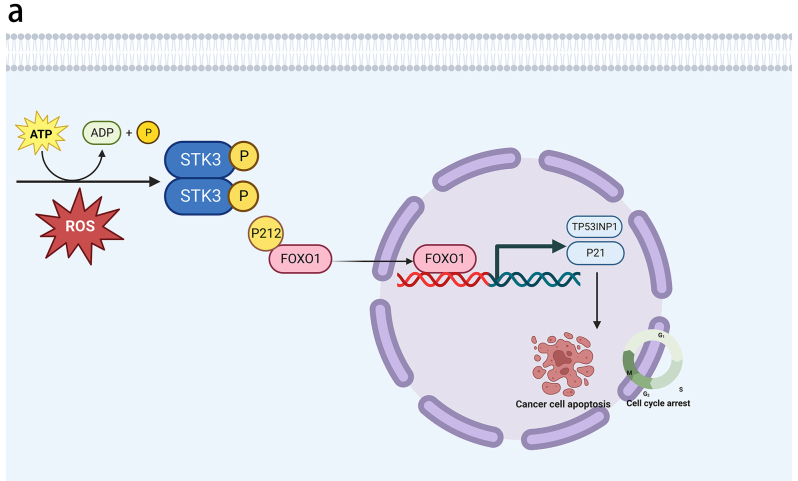
STK3 activated FOXO1-TP53INP1/P21 axis in ESCC cell lines and induced cell death. (**a**) Our study proposes a model of STK3 autophosphorylation in response to oxidative stress. The resulting p-STK3/4 complex regulates FOXO1 phosphorylation at the Serine 212 site and triggers its nuclear accumulation, leading to the transcriptional upregulation of TP53INP1 and P21. This pathway activation induces cell apoptosis and cell cycle arrest in ESCC

This study proves that STK3 kinase is an anti-tumor protein in both in vivo and in vitro ESCC. Upregulation of p-STK3/4 was observed to cause the phosphorylation of FOXO1 at Serine^212^ and the accumulation of FOXO1 in the nucleus, leading to the transcription of downstream genes TP53INP1 and P21 (Fig. 8a).

## Discussion

In the canonical Hippo pathway, STK3 triggers the activation of downstream components while simultaneously restraining YAP1/TEAD activity. A dearth of STK3/4 expression has been linked with immunodeficiency syndrome, underscoring both kinases’ critical roles in regulating immune cell functions such as adhesion, migration, proliferation, and apoptosis [23, 24]. Our study builds on this knowledge and reveals that cellular ROS can activate STK3 autophosphorylation. After overexpressing STK3 and p-STK3/4, we observed a decrease in ESCC cell proliferation and migration and an increase in cell apoptosis and growth stagnation.

Recent studies demonstrate that STK3 acts as a tumor suppressor, restricting tumor size and metastasis in the hepatic cell cancer [11], pancreatic duct cancer [25], breast cancer [26], and colorectal cancer [27, 28] while exhibiting malignant potential in the gastric cancer [29] and prostate cancer [30]. STK3/4 also serves as conserved regulators of apoptosis and autophagy. Doxorubicin (DOX) chemotherapy triggers the upregulation of p-STK3/4, which effectively inhibits YAP phosphorylation. As a result, this leads to mitochondrial damage, decreased cell viability, and enhanced apoptosis becoming evident [31]. LC3 is phosphorylated at threonine 50 by STK3/4, and the loss of p-LC3thr50 impairs the fusion of autophagosomes with lysosomes, preventing autophagy [32]. As aforementioned, other upstream kinases could also phosphorylate LATS1/2, and while STK3 plays a critical role in the Hippo pathway, p-STK3/4 might be dispensable for pathway activation. This reminds us that STK3 may have another mechanism in inhibiting cancer cell proliferation and migration independent of Hippo. We focused on the STK3 kinase and first observed that STK3 expression is high in ESCC tissue and cell lines. Additionally, our analysis of overall survival based on the TCGA database revealed that patients with low STK3 expression had a worse prognosis than those with high STK3 expression.

Autophosphorylation of STK3/4 can be induced by various stimuli, including apoptotic and stress stimuli, radiation, hydrogen peroxide, and cytokine signaling [33]. Previous studies have shown that external stimuli such as hydrogen peroxide, a-TOS, hypoxia condition, or Piperlongumine (PL) can induce STK3 activation and autophosphorylation [34–36], and a recent study also demonstrated that the NAC treatment could attenuate the STK3/4 activation [37]. Reactive oxygen species have a vital regulatory function in several biological processes, including cell cycle regulation, apoptosis, cellular senescence, autophagy, and metabolism [38]. In this study, we utilized H_2_O_2_ to induce the autophosphorylation of STK3. Furthermore, we employed NAC as a means to reverse this effect, aiming to gather further evidence regarding the activation conditions of the kinase. Verifying the activation condition of STK3 in vitro is of great significance for the exploration of therapeutic approaches for esophageal squamous cell cancer.

ROS can induce reprogramming and damage signals on cellular biological processes, presenting an opportunity to inhibit tumor progression by exploiting the differential sensitivity of specific tumor cells to oxidative stress [39]. Numerous studies have shown that ROS can trigger cell death programs and enhance medication or radiation efficacy to prevent ESCC tumor progression [40, 41]. In contrast, the detoxification of ROS can modulate metabolic reprogramming and promote cell proliferation [42]. Our study demonstrated that prolonged and steady ROS stimulation significantly increased the autophosphorylation of STK3, which facilitates its inhibitory function on tumor progression, providing evidence that the phosphorylation level determines the biological activity of STK3. We then conducted various assays to confirm the tumor suppressor role of STK3 in ESCC. Our study verified that H_2_O_2_ could cause an increased cellular ROS level and autophosphorylation of STK3 in ESCC, which may correlate with ATP levels. We also found that upregulation of p-STK3/4 is associated with increased inhibition of tumor progression. Currently, we can only measure the level of p-STK3/4 due to the limitations of the antibody, which means that we cannot determine the expression level of p-STK3 alone. However, in the future, we plan to investigate whether knocking down STK3 or STK4 can reveal any differences in their respective roles in ESCC, which will help us better understand these proteins’ specific functions.

While STK3 traditionally plays a canonical role in the Hippo pathway, its non-canonical function has recently been uncovered. G.W. Won et al. showed that STK3 phosphorylates Runx2, inhibiting its transcriptional activity during the osteoblast differentiation [43]. STK3/4 also activates Rac GTPase, promoting the assembly of the TRAF6-ECSIT complex, which is crucial for immune T cells to resist bacterial infections [44]. In cancer research, Raf-1 and Akt can interact with STK3 kinases to inhibit STK3 dimerization with RASSF1A, thus promoting cell proliferation, transformation, and survival [45]. Despite Hippo pathway kinase such as TAOK1-3 interacting with STK3, amounts of factors can further regulate the expression of STK3 and its PTM pattern in a complex way. It has been demonstrated that FGFR4 could phosphorylate the Y433 residue site of STK4, ultimately leading to the inhibition of the p-STK3/4 autoactivation [46]. The Merlin/Kibra complex plays a crucial role as a scaffold that anchors the Hippo-associated Mst and Lats kinases to the cell membrane [47, 48]. Another research showed that Fascin regulates melanoma tumorigenesis and cancer cell stemness through inhibition of the Hippo. Fascin interacts with the kinase domain of MST2, thereby impeding its homodimer formation and kinase activity [49].

The Forkhead box O (FOXO) family of transcription factors, consisting of FOXO1, FOXO3, FOXO4, and FOXO6, are mammalian counterparts of C. elegans Daf-16 and have emerged as an essential family of transcription factors that regulate the expression of genes involved in various cellular processes such as apoptosis, autophagy, DNA damage repair, cell differentiation, and glucose metabolism [50–52]. FOXO1 has been demonstrated to possess anti-tumorigenic properties in cancer cells, primarily by inducing apoptosis, cell cycle arrest, and autophagy at the transcriptional level [53, 54]. ROS regulates the function of FOXO1 through upstream signaling that activates ROS-sensitive FOXOs to maintain cellular homeostasis, acting as tumor suppressors that control the cell cycle [55]. The function of FOXO1 is also significantly influenced by PTMs. Phosphorylation, in particular, is a critical mechanism for regulating FOXO1 and FOXO3 functions, and it’s reported that STK4 plays a significant role in this process. When FOXO1/FOXO3 is phosphorylated at Ser212/Ser207 in response to oxidative stress, it accumulates in the nucleus and enhances biological functions [19, 34, 35, 56]. Negative regulators such as ERK1/2, AMPK, and AKT phosphorylate FOXO1 at Ser^23^, Ser^256,^ and Ser^310^, while positive regulators like JNK and STK4 can enhance FOXO1 transcriptional capability and regulate FOXO1 distribution in cells by interrupting negative regulation [34]. In ESCC research, FOXO1 can be acetylated, which increases its binding at the IL7R promoter, suppresses IL7R transcription, and inhibits the tumorigenic role of the IL7R [57]. In addition, FOXO1 has been found to drive the polarization of M0 macrophages and the infiltration of M2 macrophages into the TME, resulting in a worse prognosis in ESCC patients, as reported by Wang et al. [58]. In this study, we investigated the interaction between FOXO1 and STK3 kinase. We found positive evidence that STK3 overexpression could enhance FOXO1 transcriptional function by phosphorylating FOXO1 at the serine 212, which is competitive with the serine 256 site. Through rescue assays, we provided evidence that STK3 mediates FOXO1’s regulation of biological behavior in ESCC cells by activating transcription.

Furthermore, we conducted RNA sequencing analysis in siSTK3- and siFOXO1- ESCC cell lines and identified TP53INP1 and P21 as critical genes significantly downregulated in the si-STK3 and si-FOXO1 groups. We confirmed these proteins’ proapoptotic and antiproliferative functions in ESCC cell lines via cell assays. TP53INP1 and P21 are crucial proteins with pro-apoptotic and anti-proliferative properties associated with the p53 signaling pathway. TP53INP1 acts as a classic tumor suppressor that is regulated by upstream proteins. Studies have demonstrated a significant reduction or loss of TP53INP1 expression in the ESCC [59], and low expression levels of TP53INP1 have been linked to a poor prognosis in various cancers. Additionally, loss of TP53INP1 has been found to promote metastasis in early-stage hepatocellular carcinoma cells through the DUSP10 phosphatase-mediated activation of the ERK pathway [60]. The miRNAs also regulate TP53INP1 expression. For example, L. Han et al. found that TP53INP1 is a negative factor of miR-106a, which significantly reversed the promoting effect of miR-106a on lung adenocarcinoma migration and invasion [61]. P21, on the other hand, is stabilized by STK3/4 in a JNK- and Thr57-dependent manner, and RASSF6 activates the Hippo pathway and STK3/4 with the upregulating P21, cleaved caspase 3, and cytochrome c expression simultaneously [62, 63]. The RASSF1A-STK4 pathway also mediates FOXO3a transactivation on P21 and P27 (CDKN1B) [64]. Furthermore, the downregulation of FOXO1 has been found to suppress P21 and P27 expression, thereby promoting proliferation and tumorigenesis in the human breast cancer [65].

The results of the current study present compelling evidence regarding the significant involvement of STK3 kinase in regulating cellular proliferation and apoptosis in ESCC. Specifically, our findings suggest that the upregulation of STK3 and its activation form (p-STK3/4) promotes the phosphorylation of FOXO1^Ser212^, thereby augmenting the transcriptional activity of TP53INP1 and P21. These downstream effector molecules are pivotal in promoting apoptosis and suppressing proliferation in ESCC cells. Notably, the discovery of the non-canonical function of STK3 kinase, highlights the intricate and dynamic signaling networks integral to understanding cancer biology.

## Conclusion

In conclusion, our study first demonstrated the potential tumor-suppressive role and the relative mechanism of STK3 in ESCC. Moreover, transcriptional factor FOXO1 was identified as the downstream effector of STK3, affecting the TP53INP1 and P21 expression which are important in pro-apoptosis and anti-proliferation. Although there are more in-depth mechanisms and prognostic roles for STK3 in ESCC that need to be investigated in the future, our findings first provide an overall description of STK3 kinase as a potential tumor suppressor gene in ESCC.

### Electronic supplementary material

Below is the link to the electronic supplementary material.

Supplementary Material 1

Supplementary Material 2

## Data Availability

The datasets used and/or analyzed during the current study are available from the corresponding author upon reasonable request.

## Abbreviations

STK3: Serine/Threonine Kinase 3
STK4: Serine/Threonine Kinase 4
FOXO1: The Forkhead box O1
TP53INP1: Tumor protein p53-inducible nuclear protein 1
CDKN1A (P21): Cyclin-dependent kinase inhibitor 1
ESCC: Esophageal Squamous cell carcinoma
ROS: Reactive Oxygen Species
PTM: Post-transcriptional modification
shRNA: short hairpin RNA
siRNA: small interfering RNA

## Declaration

### Animal studies

All institutional and national guidelines for the care and use of laboratory animals were followed.
