# Supplementary material for: STK3 kinase activation inhibits tumor proliferation through FOXO1-TP53INP1/P21 pathway in esophageal squamous cell carcinoma
Source: Cell Oncol (Dordr). 2024 Mar 4;47(4):1295–314. doi: 10.1007/s13402-024-00928-8 (PMC11322239; doi:10.1007/s13402-024-00928-8)
Supplement: Supplementary file 1 — Supplementary Material 1 [file 13402_2024_928_MOESM1_ESM.docx]

STK3 kinase activation inhibits tumor proliferation through FOXO1-TP53INP1/P21 pathway in esophageal squamous cell carcinoma

Ziying Zhao ^1^ Yuan Chu ^1.^ Anqi Feng ^1^  Hao Wu ^1^ Zhaoxing Li ^1^ Mingchuang Sun ^1^ Li Zhang ^1^

Tao Chen* ^1^  Meidong Xu* ^1^

**Affiliation:** ^1^ Endoscopy Center, Department of Gastroenterology, Shanghai East Hospital, School of Medicine, Tongji University, Shanghai, 200120, China.

E-mail for Corresponding authors:

Tao Chen*: [chentao@tongji.edu.cn](mailto:chentao@tongji.edu.cn)

Meidong Xu* : [1800512@tongji.edu.cn](mailto:1800512@tongji.edu.cn)

SUPPLEMENTARY MATERIALS

Figure S1 Supplementary statistical results.

Figure S2 Moderate concentration of H_2_O_2_ induces the upregulation of cellular ROS level.

Figure S3 Supplementary results on STK3 phosphorylate FOXO1 at serine 212 site.


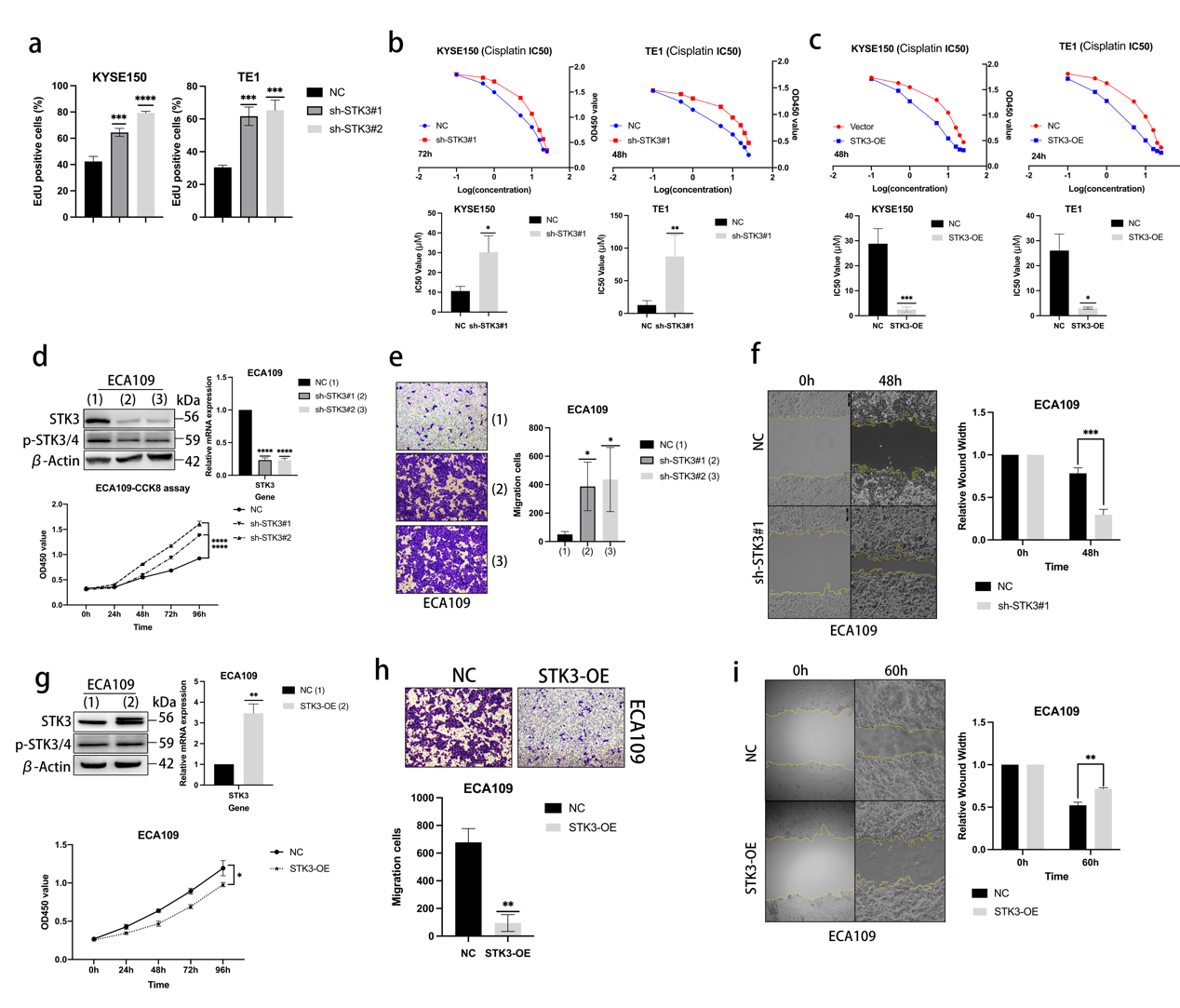


***Figure.S1*** *Supplementary statistical results.* (a) Statistical results of the EdU assay in KYSE150 and TE1 cell lines. (b-c) KYSE150 and TE1 cells stably expressing shRNA-STK3 or STK3-OE were seeded on 96-well, and cisplatin was diluted in PBS according to the concentration gradient. The 450nm OD value was measured after the 1-hour incubation of CCK-8. Statistical results of IC50 value in shRNA-STK3 group in KYSE150 and TE1 cell lines. (* p < 0.05, ** p < 0.01, *** p < 0.001, **** p < 0.0001.). (d) ECA109 cells stabling expressing shRNA specific to STK3 were cultured for at least 24 hours, and the cell lysates were subjected to western blotting and Q-PCR. The cell viability was also examined by CCK8 assay. (e-f) ECA109 cells stabling expressing shRNA-STK3 were subjected to transwell and wound scratch assays. (g) STK3 overexpression in ECA109 cells. The cell lysates were subjected to western blotting and Q-PCR. The cell viability and migration were examined by CCK8 assay. (h-i) The migration capability of ECA109 cells stably expressing shSTK3 transwell assay and wound scratch assay.


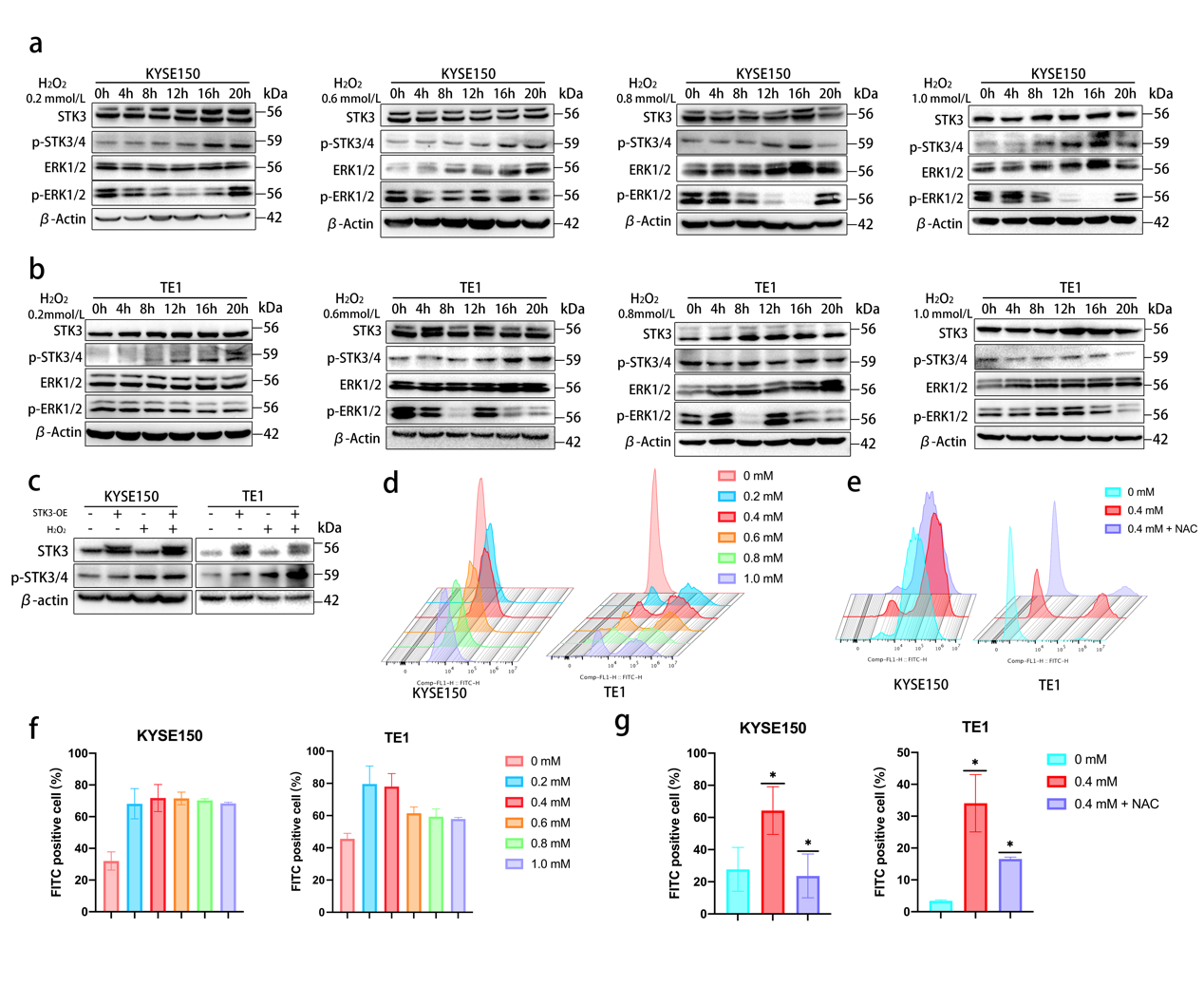


***Figure.S2*** *Moderate concentration of H_2_O_2_ induces the upregulation of cellular ROS level.* (a-b) The concentration of H_2_O_2_ was set from 0mM to 1.0mM, and KYSE150 and TE1 cells were treated with different concentrations between 0 and 20 hours at the 4-hour interval. The cell lysates were subjected to immunoblotting using specific antibodies. (c) KYSE150 and TE1 cells stably overexpressing STK3 were treated by 0mM or 0.4mM H_2_O_2_. The cells were cultured for 20 hours, and the cell lysates were subjected to western blotting using specific antibodies. (d) The KYSE150 and TE1 cells were treated with different concentrations of H_2_O_2_ for 20 hours. The cells were collected and made into suspension according to the manufacturer’s instructions, and then the cell suspension was subjected to the flow cytometer. (e) The KYSE150 and TE1 cells were treated with 0.4 mM H_2_O_2_ with or without NAC. The cells were processed and analyzed as aforementioned. (f) The results gathered from two independent experiments showed intracellular ROS levels in the concentration gradient model in KYSE150 and TE1 cell lines. (g) The statistical results of the intracellular ROS levels in NAC rescue experiments. The data were presented from three independent experiments in duplicate (* p < 0.05).

*
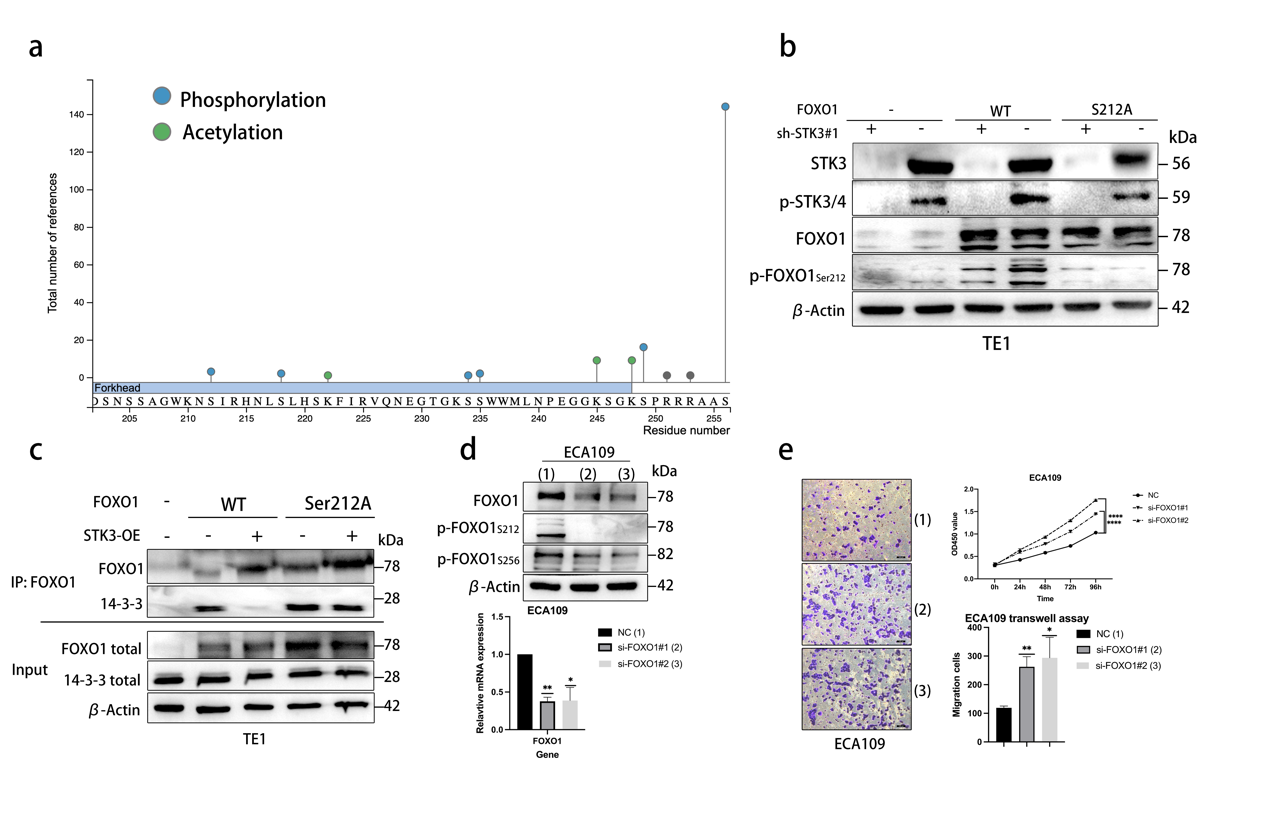
*

***Figure.S3*** Supplementary results on *STK3 phosphorylate FOXO1 at serine 212 site.*

(a) The online database showed the amino acid sequence of the fox head domain and the possible phosphorylation or acetylation site in FOXO1 (<https://www.phosphosite.org/homeAction.action>). (b) The TE1 cells with STK3 deletion were transfected with siRNA-FOXO1#1 or pVL2-CMV-FOXO1^S212A^, and the cell lysates were subjected to western blotting using specific antibodies. (c) The TE1 cells stably overexpressing STK3 were transfected with siRNA-FOXO1#1 or pVL2-CMV-FOXO1^S212A^ cultured in 10 cm plates cell lysates were subjected to immunoprecipitation using an anti-FOXO1 antibody. The protein samples were then subjected to western blotting. (d-e) The ECA109 cells were transfected by siRNA specific to FOXO1. The knockdown efficiency was evaluated by western blotting and Q-PCR. The cell viability and migration were examined by CCK8 assay and transwell assay (* p < 0.05, ** p < 0.01, *** p < 0.001.).
