# Supplementary material for: STK3 kinase activation inhibits tumor proliferation through FOXO1-TP53INP1/P21 pathway in esophageal squamous cell carcinoma
Source: Cell Oncol (Dordr). 2024 Mar 4;47(4):1295–314. doi: 10.1007/s13402-024-00928-8 (PMC11322239; doi:10.1007/s13402-024-00928-8)
Supplement: Supplementary file 2 — Supplementary Material 2 [file 13402_2024_928_MOESM2_ESM.docx]

STK3 kinase activation inhibits tumor proliferation through FOXO1-TP53INP1/P21 pathway in esophageal squamous cell carcinoma

Ziying Zhao ^1^ Yuan Chu ^1.^ Anqi Feng ^1^  Hao Wu ^1.^ Zhaoxing Li ^1.^ Mingchuang Sun ^1.^ Li Zhang ^1.^

Tao Chen* ^1.^ Meidong Xu* ^1^

**Affiliation:** ^1^ Endoscopy Center, Department of Gastroenterology, Shanghai East Hospital, School of Medicine, Tongji University, Shanghai, 200120, China.

E-mail for Corresponding authors:

Tao Chen*: [chentao@tongji.edu.cn](mailto:chentao@tongji.edu.cn)

Meidong Xu* : [1800512@tongji.edu.cn](mailto:1800512@tongji.edu.cn)

SUPPLEMENTARY MATERIALS

Table S1 Sequences ﻿of specific Plasmids and lentivirus

Table S2 ﻿Primer sequences ﻿used in Quantitative PCR

Table S3 ﻿Primer sequences ﻿used in ChIP PCR

*Table S1 Sequences ﻿of specific Plasmids and lentivirus*

| Gene | Plasmids | S | AS |
| --- | --- | --- | --- |
| STK3 | shRNA#1 | GAGCUAAAGUUGCAGACAATT | UUGUCUGCAACUUUAGCUCTT |
|  | shRNA#2 | CCUUGCCUGUUGACUUUGUTT | ACAAAGUCAACAGGCAAGGTT |
| FOXO1 | siRNA#1 | GGUCCAAGGAAAGUUUAUATT | UAUAAACUUUCCUUGGACCTT |
|  | siRNA#2 | GCAGCCUUGUUUGAUUUAUTT | AUAAAUCAAACAAGGCUGCTT |
| TP53INP1 | siRNA#1 | CAGAAGAAGAAGAAGAAGATT | UCUUCUUCUUCUUCUUCUGTT |
|  | siRNA#2 | CCGUGGGACUGAUGAAUUATT | UAAUUCAUCAGUCCCACGGTT |
| P21 | siRNA#1 | CAGGCGGUUAUGAAAUUCATT | UGAAUUUCAUAACCGCCUGTT |
|  | siRNA#2 | GAUGGAACUUCGACUUUGUTT | ACAAAGUCGAAGUUCCAUCTT |

*Table S2 ﻿Primer sequences ﻿used in Quantitative PCR*

| Gene | ﻿Forward primer (5'>3') | ﻿Reverse primer (5'>3') |
| --- | --- | --- |
| STK3 | TAGCACGATGTTGGAATCCGACTTG | TGGTCTTTGTACTTGTGGTGAGGTTG |
| FOXO1 | CTACGAGTGGATGGTCAAGAG | ATGAACTTGCTGTGTAGGGAC |
| TP53INP1 | CAGGTGTTGTAGACAGAGGCACTTG | GGACAGAATTGGCGGGAAGGAATAG |
| P21 | TGTCACTGTCTTGTACCCTTG | GGCGTTTGGAGTGGTAGAA |
| BIM | AAGAGTTGCGGCGTATTGGAGAC | CCACACCAGGCGGACAATGTAAC |

*Table S3 ﻿Primer sequences ﻿used in ChIP PCR*

| Gene | ﻿Forward primer (5'>3') | ﻿Reverse primer (5'>3') |
| --- | --- | --- |
| TP53INP1 (1657-1670) | GTTCTGCGCCCTTTGTGCCCC | GTAAGAGGCGGGCGAGGAAGA |
| P21 (1922-1935) | TTCCCCTACCCCATGCTGCTCCAC | AGAAAGCCAATCAGAGCCACAGCC |
